# Exposure to flame retardant chemicals on commercial airplanes

**DOI:** 10.1186/1476-069X-12-17

**Published:** 2013-02-16

**Authors:** Joseph G Allen, Heather M Stapleton, Jose Vallarino, Eileen McNeely, Michael D McClean, Stuart J Harrad, Cassandra B Rauert, John D Spengler

**Affiliations:** 1 Harvard School of Public Health, Boston, MA, USA; 2 Duke University, Nicholas School of the Environment, Durham, NC, USA; 3 Boston University School of Public Health, Boston, MA, USA; 4 University of Birmingham, School of Geography, Earth and Environmental Sciences, Birmingham, UK

**Keywords:** Flame retardants, Airplanes, Dust exposure, Hand-wipe samples

## Abstract

**Background:**

Flame retardant chemicals are used in materials on airplanes to slow the propagation of fire. These chemicals migrate from their source products and can be found in the dust of airplanes, creating the potential for exposure.

**Methods:**

To characterize exposure to flame retardant chemicals in airplane dust, we collected dust samples from locations inside 19 commercial airplanes parked overnight at airport gates. In addition, hand-wipe samples were also collected from 9 flight attendants and 1 passenger who had just taken a cross-country (USA) flight. The samples were analyzed for a suite of flame retardant chemicals. To identify the possible sources for the brominated flame retardants, we used a portable XRF analyzer to quantify bromine concentrations in materials inside the airplanes.

**Results:**

A wide range of flame retardant compounds were detected in 100% of the dust samples collected from airplanes, including BDEs 47, 99, 153, 183 and 209, tris(1,3-dichloro-isopropyl)phosphate (TDCPP), hexabromocyclododecane (HBCD) and bis-(2-ethylhexyl)-tetrabromo-phthalate (TBPH). Airplane dust contained elevated concentrations of BDE 209 (GM: 500 ug/g; range: 2,600 ug/g) relative to other indoor environments, such as residential and commercial buildings, and the hands of participants after a cross-country flight contained elevated BDE 209 concentrations relative to the general population. TDCPP, a known carcinogen that was removed from use in children’s pajamas in the 1970’s although still used today in other consumer products, was detected on 100% of airplanes in concentrations similar to those found in residential and commercial locations.

**Conclusion:**

This study adds to the limited body of knowledge regarding exposure to flame retardants on commercial aircraft, an environment long hypothesized to be at risk for maximum exposures due to strict flame retardant standards for aircraft materials. Our findings indicate that flame retardants are widely used in many airplane components and all airplane types, as expected. Most flame retardants, including TDCPP, were detected in 100% of dust samples collected from the airplanes. The concentrations of BDE 209 were elevated by orders of magnitude relative to residential and office environments.

## Introduction

The weight of combustible materials in airplanes ranges from 3000 kg to over 7000 kg in wide-body airplanes [1]. To protect the flying public and flight crew it is essential that materials within airplanes have flame retardant properties. Due to strict fire safety regulations, materials used on airplanes are subject to a battery of fire tests before being approved for use. These materials, therefore, need to be inherently fire-resistant, have a fire-resistant barrier or must incorporate flame retardant chemicals within the product. Testing of the finished product for flammability is evaluated on a performance-based standard; manufacturers of the products are not required to disclose the identity of the flame retardant chemicals used in the product to meet flammability requirements.

The history of flame retardant usage in consumer products over the past three decades includes the introduction of these chemicals, their subsequent removal or ban after determination of potential health consequences, followed by the introduction of alternative chemicals that have not been well characterized in terms of exposure or potential health effects. Polychlorinated biphenyls (PCBs) and polybrominated biphenyls (PBBs) were used as flame retardant chemicals in products until their ban (PCBs) and voluntary withdrawal (PBBs) in the 1970s due to concerns regarding their toxicity. Tris (2,3-dibromopropyl) phosphate, a mutagenic flame retardant used in children’s pajamas, was phased out in the 1970s because it was found that children absorbed the chemical after wearing flame retardant treated pajamas [2]. Use of its chlorinated analog, tris(1,3-dichloro-isopropyl)phosphate (TCDPP), was also discontinued at the time due to a study that found that the chlorinated version was similarly mutagenic [3] [TDCPP was re-introduced in other commercial products later]. Another class of flame retardants, polybrominated diphenyl ethers (PBDEs), manufactured in three commercial products – PentaBDE, OctaBDE and DecaBDE – have been used widely in consumer products from the 1980’s – 2000’s. Two of the three PBDE commercial products (PentaBDE and OctaBDE) were voluntarily phased out in the U.S. in 2005 and the third commercial product (DecaBDE) is being phased out in 2013 because of their persistence in the environment, ability to bioaccumulate, demonstrated neuro- and developmental toxicity and potential for endocrine disruption [4-6]. Several newer and commercially important flame retardant chemicals are now being incorporated into products to replace PBDEs [7,8]. These include brominated flame retardants (tetrabromobisphenol A [TBBPA], hexabromocyclododecane [HBCD]), and bis-(2-ethylhexyl)-tetrabromo-phthalate [TBPH], and chlorinated phosphates (tris(1,3-dichloro-isopropyl) phosphate [TDCPP]. Toxicological evidence suggests that these compounds may have important human health implications. For example, TDCPP was recently added to U.S. State of California’s Proposition 65 list as a chemical known to cause cancer [9], and levels in house dust were found to be associated with altered sex hormones in men [10].

Flame retardants are often used in large quantities relative to the mass of the product; PBDEs are found up to 20% by weight in consumer products used in homes [11] and TDCPP has been reported up to 9% by weight in foam [12] and 12.5% in baby products [13]. These flame retardants are used as additives and therefore can migrate from their source products. Not surprisingly, then, many studies have reported finding high concentrations of PBDEs and TDCPP in dust from homes and offices [8,14-16], and dust has been shown to be an important source of exposure for flame retardants [17].

Our understanding of exposure to flame retardant chemicals on airplanes, a micro-environment of particular interest due to the potential for high flame retardant usage, is limited to a small number of studies that were focused on PBDEs. In the first peer-reviewed paper related to airplanes, Christiannsson et al. reported that PBDE concentrations in dust obtained from airplanes that were higher than levels typically seen in homes, and they found that travelers had higher body burdens of PBDEs post-flight [18]. We recently published a study of cabin air concentrations of PBDEs on in-flight airplanes [19] and found that concentrations in airplanes were elevated compared to concentrations in U.S. and U.K. homes [20,21] and similar to concentrations found in industrial environments [22-25]. Schecter et al. measured serum concentrations from 30 flight attendants/pilots and reported no difference in serum concentrations compared to the general public (U.S.), on average, although several individuals had elevated serum concentrations [26].

The limited exposure data currently available suggest that airplanes may be a potentially relevant exposure environment for flame retardant chemicals for passengers, cleaning and flight crews, and workers installing or retrofitting cabin interiors. Additional research is needed to characterize exposure to health-relevant flame retardants and fully elucidate the source-environment-receptor exposure pathways specific to commercial airplanes. This study aimed to address one of these knowledge gaps by characterizing the dust exposure pathway for flame retardants on airplanes.

## Methods

### Airplanes

Dust samples were collected in November and December, 2010, on commercial airplanes that were parked overnight at an international airport. Identification of bromine in airplane materials was also performed on the same aircraft using x-ray fluorescence (XRF). The airplanes represented a wide range of manufacturing dates (1986 – 2008) from five manufacturers (Boeing, Airbus, Canadair Regional, McDonnell Douglas and Embraer).

### Dust sample collection

Two types of dust samples were collected on each of 19 airplanes (1 airplane was re-sampled), yielding a total of 40 dust samples. On each plane, one dust sample was obtained by vacuuming the carpet, with a second sample obtained by vacuuming the air return grilles located near the floor on the wall of the plane. Dust samples were collected on airplanes using a standardized collection protocol based on previously published methods [14,27]. Briefly, a cellulose extraction thimble was fit into a vacuum crevice tool and secured using a rubber O-ring. The sampling tool was then connected to a canister vacuum and researchers collect dust vacuuming either the carpet or vents. After dust collection, the thimbles were wrapped in aluminum foil, sealed in polyethylene zip bags and stored at −4°C. Sample equipment was cleaned in a 1% solution of detergent and hot water between sample collection to prevent cross contamination. Prior to analysis, dust was sieved to <500 μm.

### Dust laboratory analysis

Dust samples (approximately 300 mg) were extracted and analyzed for PBDEs, alternate BFRs and OPFRs using our previously published method [7] with slight modifications. Dust samples were first spiked with two internal surrogate standards, 4-fluoro-2,3,4,6-tetrabromodiphenylether (FBDE 69) and ^13^C labeled decabromodiphenyl ether (^13^C BDE 209). Dust samples were extracted with 50:50 hexane: dichloromethane (DCM) using pressurized solvent extraction (ASE 300, Dionex). A temperature of 100°C and a pressure of 1500 psi was used in the ASE system. The final extract was reduced in volume to approximately 1 mL using an automated nitrogen evaporation system (Turbo Vap II, Zymark Inc.). Extracts were then purified by elution through a Florisil solid-phase extraction cartridge utilizing the method reported by [28]. A first fraction eluted with hexane contained the PBDEs, BTBPE, syn- and anti- Dechlorane Plus (DP), HBB, TBB, and TBPH. The second fraction eluted with ethyl acetate contained the OPFRs. The two fractions were separately concentrated to 1 mL in volume, spiked with a recovery standard, ^13^C-2,2’,3,4,5,5’-hexachlorodiphenylether (CDE 141) and analyzed by GC/MS. After GC/MS analyses, the two fractions were combined and analyzed by LC/MSMS for isomer specific HBCDs.

### Dust sample analysis

All F1 samples were analyzed using gas chromatography mass spectrometry operated in electron capture negative ionization mode (GC/ECNI-MS), similar to our previous analysis of dust samples [7]. A 0.25 mm (I.D.) × 15 m fused silica capillary column coated with 5% phenyl methylpolysiloxane (0.25 μm film thickness) was used for the separation of BDE congeners. Pressurized temperature vaporization (PTV) injection was employed in the GC. The inlet was set to a temperature of 50°C for 0.3 minutes and then a 700°C/min ramp to 275°C was employed to efficiently transfer the samples to the head of the GC column. The oven temperature program was held at 40°C for 1 min followed by a temperature ramp of 18°C /min to 250°C, followed by a temperature ramp of 1.5°C /min to a temperature of 260°C, followed by a final temperature ramp of 25°C/min to 300°C which was held for an additional 20 min. The transfer line temperature was maintained at 300°C and the ion source was held at 200°C. PBDEs, HBB, and BTBPE, were quantified by monitoring bromide ions (m/z 79 and 81). Due to co-elution issues between BDE 99 and TBB, BDE 99 was also measured using GC/MS operated in electron impact mode (GC/EI-MS) by monitoring ion fragments 484 and 404. ^13^C BDE-209 was monitored through m/z 494.6 and 496.6. TBB was quantified using ion fragments (m/z) 357 (Quantitative) and 471 (Qualitative) while TBPH was quantified using ion fragments (m/z) 463 (Quant) and 515 (Qual). Dechlorane plus isomers were quantified using *m/z* 652 and 654. All F2 extracts were spiked with 100 ng of deuterated TDCPP (dTDCPP) and analyzed using GC/MS operated in electron impact mode (GC/EI-MS). The GC conditions were the same as listed above. The following ions were monitored for dTDCPP and TDCPP, respectively, 394/392 and 381/383. After GC/MS analysis, the two fractions were recombined, blown to dryness, spiked with 100 ng each of ^13^C labeled HBCD isomers (alpha, beta and gamma), and then analyzed by liquid chromatography tandem mass spectrometry using our previously published method [29].

### Quality assurance for dust analysis

As part of our quality assurance criteria we examined levels of these specific BFR analytes in laboratory blanks (n=3), and a dust Standard Reference Material (SRM 2585, National Institute of Standards & Technology, Gaithersburg, MD; n=3). Low levels of several analytes were detected in laboratory blanks. BDEs 28, 47, 99, 100, 153, 154, HBCD, TBB, TBPH and TDCPP were detected in laboratory blanks and ranged from 0.04 ± 0.01 ng for BDE 28 to 5.0 ± 4.0 ng for BDE 99; however, these levels were much lower than levels measured in the samples (<1% typically). BDE 209 was detected at higher levels in lab blanks, averaging 25.9 ± 19.9 ng, and is likely due to carryover from the dust samples during the extraction on the ASE system. Due to the high levels of BDE 209 in all the samples, extracts were diluted 100 fold, re-spiked with ^13^C BDE 209, and re-run for more accurate measurements. All sample measurements were blank corrected by subtracting the average level measured in the laboratory blanks. Method detection limits were calculated by taking three times the standard deviation of the blank levels. As a further quality control step, we analyzed SRM 2585. Recoveries for all PBDEs (except BDE 209) were at 87 ± 18%. Higher recoveries for BDE 209 in SRM 2585 were observed, ranging from 139–339% and were likely due to carryover issues of BDE 209 from the dust extracts as very high levels of BDE 209 were observed in these samples. Therefore, these measurements of BDE 209 should be interpreted cautiously. Measurements of BTBPE, HBCD, TBB and TBPH in SRM 2585 ranged from 73 to 121% of those reported by [30].

### Source identification by XRF

Bromine concentration in products, as determined by x-ray fluorescence, have been previously shown to be a useful indicator of PBDE concentration in consumer products [11]. To identify materials on airplanes that were likely to contain PBDEs, we used a portable XRF analyzer (Innov-X, Woburn, MA) to quantify bromine concentrations in materials aboard the 19 airplanes. Over 200 measurements were recorded (between 5 and 23 samples were collected per airplane). Measurements were obtained by placing the XRF analyzer directly on the surface of the material and obtaining a 10–20 second reading. Materials sampled include: seats, carpets, walls, overhead bins, pillows and other accessible items within the cabin interior. XRF penetration depth varies depending on the material being sampled, and is in the millimeter to centimeter range for light plastics, and a few millimeters for heavier plastics.

### Quality assurance for XRF measurements

Several QA/QC parameters were evaluated to assess the accuracy and precision of the XRF measurements. First, the analyzer was calibrated by the manufacturer immediately prior to use. Second, a calibration curve for bromine was established by comparing bromine measurements made using the XRF analyzer and corresponding PBDE measurements by GC/MS from 22 products. This calibration curve was further validated by measuring bromine concentrations in two NIST standards. Third, repeat measurements were collected in the field and showed strong agreement.

### Hand-wipes sample collection

In June, 2011, a total of 20 hand-wipe samples were collected from 10 individuals who had just completed a cross-country (USA) flight; the airplanes were not the same airplanes that the dust samples were collected on. Two samples were collected per participant (palms and back of hands) and nine of the 10 participants were flight attendants (the 10^th^ participant was a researcher who completed the same flight with one group of flight attendants enrolled in the study). The palm samples were intended to represent dust accumulation from touching surfaces and from the air, and the samples from the back of hands represent dust accumulation from the air. Samples were collected at a hotel after disembarkation and travel to the hotel via taxi. Subjects were instructed to avoid washing hands for at least one hour before the end of the flight until sampling.

Hand-wipes were collected using a method first employed in the National Human Exposure Assessment Survey (NHEXAS) for pesticides and volatile organic compounds (VOCs) [31], and later adapted to PBDEs [32]. A sterile gauze pad (7.6 cm × 7.6 cm) was placed in a clean aluminum tray containing 3.0 ml of isopropyl alcohol. Once the gauze pad is soaked, field technicians wiped the entire surface area of both palms of the study participant, from wrist to fingertips. This was then repeated for the back of the hands. Completed hand-wipe samples were kept separate and placed in pre-cleaned 50 ml glass centrifuge tubes and stored at −20°C until analysis. Hand-wipe sampling protocols were approved by the Institutional Review Board at the Harvard School of Public Health.

### Hand-wipes laboratory analysis

Each sample was transferred to a 66 mL accelerated solvent extractor (ASE) cell and the void volume filled with Hydromatrix (Varian Inc., U.K.). Each cell was spiked with 50 ng of labeled ^13^C-BDE 47; 12.5 ng each of ^13^C-BDE 99, 153; and 25 ng of ^13^C-BDE 209 as surrogate standards. Extraction was performed using pressurized liquid extraction (ASE 350, Dionex Europe, U.K.) using hexane/dichloromethane (1:1, v/v) at 45°C and 1500 psi with a heating time of 5 min, static 4 min, purge time 90 s, flush volume 50%, with three static cycles. The extracts were concentrated to 0.5 mL using a Zymark Turbovap II then purified by loading onto SPE cartridges filled with 8 g of precleaned acidified silica (44% concentrated sulfuric acid, w/w). The analytes were eluted with 30 mL of hexane/dichloromethane (1:1, v/v). The eluate was evaporated to dryness under a gentle stream of nitrogen, then reconstituted to 50 *μ*L with 2.5 *μ*L of ^13^C-BDE 100 (4000 ng/mL) used as a recovery standard, plus 47.5 *μ*L of toluene.

Laboratory analysis was conducted with a modified version of a previously published method [33]. In summary, target PBDEs (47, 85, 99, 100, 153, 154, 183 and 209) were separated using a dual pump Shimadzu LC-20AB Prominence liquid chromatograph (Shimadzu, Kyoto, Japan) equipped with a SIL-20A autosampler, a DGU-20A3 vacuum degasser, and a Varian Pursuit XRS3 (Varian, Inc., Palo Alto, CA) C18 reversed phase analytical column (250 mm x 4.6 mm i.d., 3 *μ*m particle size). A mobile phase program based upon (mobile phase A) 1:1 methanol/water and (mobile phase B) 2:8 toluene/methanol at a flow rate of 0.4 mL/min was applied for elution of the target compounds; starting at 85% (mobile phase B), then increased linearly to 100% (mobile phase B) over 20 min, and then held for 10 min. The column was equilibrated with 85% (mobile phase B) for 5 min between runs. Mass spectrometric analysis was performed using a Sciex API 2000 triple quadrupole mass spectrometer (Applied Biosystems, Foster City, CA) equipped with an APPI ion source operated in negative ion mode.

### Quality assurance for hand-wipes analysis

For every five samples, one method (laboratory) blank was analyzed, consisting of the extraction of precleaned hydromatrix, treated identically to the samples. Concentrations of PBDEs in blanks never exceeded 1% of the concentration in a sample from the same batch and data were thus not blank-corrected. Ongoing method performance was assessed through the regular analysis of matrix spike samples (n=3), each consisting of a blank hand-wipe spiked with 20 ng of BDE 47, 85, 99, 100, 153, 154, 183 and 209 native compounds, and treated identically to the samples. Two samples were excluded from the analysis due to unacceptably low surrogate standard recoveries (< 25%).

### Statistical analysis

Data handling and statistical analyses were performed using SAS (v. 9.1.3, Cary, NC). Descriptive statistics were compiled by substituting ½ the detection limit for values less than the limit of quantification (LOQ). Spearman correlation coefficients (r) were calculated to evaluate associations between variables, and Wilcoxon rank sum tests were used to aid in the interpretation of potential differences between flame retardant concentrations in different sample types and different sample locations. Statistical significance was determined at the α=0.05 level.

## Results & discussion

### Flame retardant concentrations in airplane dust

Flame retardant concentrations in airplane dust from this study are presented alongside comparative data from studies of airplanes, U.S. homes and offices in Table 1. For PBDEs, data are presented for the 13 of 24 congeners detected in greater than 75% of samples. These major congeners of the three commercial mixtures were detected in nearly all of the dust samples collected. Concentrations of BDEs 47, 99, 100, 153 and 154, major congeners in the PentaBDE commercial mixture, were similar to the measured concentrations in another study of airplanes during intercontinental flights from Sweden [17] and concentrations found in U.S. homes [14], yet slightly elevated compared to concentrations in U.S. offices [16]. Concentrations of BDE 183, the major congener in the OctaBDE commercial product, were similar to the other study of airplane dust but elevated compared to homes and offices. For BDE 209, which is the primary congener of the DecaBDE commercial product, the median dust concentrations measured in this study were an order of magnitude higher than previously found in airplanes, and several orders of magnitude higher than what is typically found in U.S. homes and offices. An important difference in the dust samples from airplanes reported for the Swedish study and this study is the sample collection method. In this study, we collected dust from the carpet and air supply return vents near the floor using a standardized collection protocol. The method of dust sample collection in the Swedish study is not fully described beyond the notation that there was limited visible dust except for the bathroom vents. Differences in BDE 209 concentrations between the two studies may be attributable to the type of dust collected. Dust collected in the carpet may be enriched in BDE 209 due to the proximity to a source and greater collection of larger, settled dust particles.

**Table 1:** Concentrations of flame retardants in dust (ng/g) on commercial airplanes in comparison to other indoor environments (n=40)

	**Airplanes (this study)**				**Airplanes**^**1**^		**Homes**^**2,3,4**^		**Offices**^**5**^	
**Flame retardant**	**Sample location**	**Pct detect**	**Median**	**Range**	**Median**	**Range**	**GM/Median**	**Range**	**GM/Median**	**Range**
BDE28, 33	Floor	100%	54	(5.3 - 270)	199	(12–2800)	16	(1.6 - 120)	8	(<0.4 - 210)
	Vent	95%	33	(0.8 - 140)						
BDE47	Floor	100%	950	(280–17000)	4100	(0.4 - 230000)	1900	(450–17000)	700	(37–19000)
	Vent	100%	3500	(230–19000)						
BDE49	Floor	84%	40	(0.3 - 6300)	--	--	30	(0.3 - 3700	19	(<0.4 - 610)
	Vent	68%	65	(0.4 - 790)						
BDE66	Floor	100%	45	(9.0 - 1500)	--	--	17	(0.2 - 290)	9	(<0.2 - 500)
	Vent	95%	230	(0.7 - 620)						
BDE75	Floor	100%	370	(34–47000)	--	--	9	(1.3 - 75)	40	(<0.4 - 230)
	Vent	100%	310	(27–18000)						
BDE85, 155	Floor	95%	76	(11–2900)	--	--	120	(18–1100)	50	(<0.2 - 3100)
	Vent	100%	200	(14–2200)						
BDE99	Floor	100%	950	(330–37000)	4000	(160–290000)	2500	(330–25000)	920	(<0.4 - 33000)
	Vent	100%	4200	(360–35000)						
BDE100	Floor	100%	180	(43–8900)	1100	(36–180000)	440	(71–4300)	200	(13–8700)
	Vent	95%	630	(45–6100)						
BDE138	Floor	100%	20	(5.9 - 210)	--	--	21	(0.1 - 240)	18	(1.6 - 960)
	Vent	68%	62	(0.3 - 680)						
BDE153	Floor	100%	230	(65–5300)	590	(17–23000)	230	(28–2100)	140	(11–6000)
	Vent	100%	630	(33–4700)						
BDE154	Floor	100%	120	(33–4700)	670	(15–63000)	180	(27–2100)	120	(7.6 - 5200)
	Vent	95%	280	(24–3400)						
BDE183	Floor	100%	620	(200–5500)	490	(3.5 - 190000)	28	(1.7 - 230)	80	(15–13000)
	Vent	100%	390	(76–9100)						
BDE209	Floor	100%	495000	(210000–2100000)	22000	(440–190000)	4500	(790–180000)	4200	(910–110000)
	Vent	100%	473000	(190000–2600000)						
BTBPE	Floor	100%	330	(30–48000)	--	--	21	(1.4 - 950)	--	--
	Vent	100%	1300	(160–25000)						
*anti*-Dechlorane Plus	Floor	100%	330	(92–4200)	--	--	--	--	--	--
	Vent	100%	300	(31–9600)						
*syn*-Dechlorane Plus	Floor	100%	110	(40–9500)	--	--	--	--	--	--
	Vent	100%	160	(34–2200)						
HBB	Floor	95%	100	(18–540)	--	--	--	--	--	--
	Vent	53%	45	(23–210)						
α-HBCD	Floor	84%	2300	(4.7 - 290000)	--	--	80	(17–1800)	--	--
	Vent	84%	1600	(17–32000)						
β-HBCD	Floor	74%	310	(1.2 - 75000)	--	--	28	(6–300)	--	--
	Vent	63%	230	(0.8 - 11000)						
γ-HBCD	Floor	100%	4500	(130–700000)	--	--	300	(79–2000)	--	--
	Vent	95%	7600	(99–59000)						
Total HBCD	Floor	100%	7600	(180–1100000)	--	--	170	(<2 - 2800)	--	--
	Vent	100%	10000	(370–97000)						
TBB	Floor	100%	350	(200–3000)	--	--	840	(<450 - 75000)	--	--
	Vent	100%	740	(300–5000)						
TBPH	Floor	100%	640	(400–1600)	--	--	650	(<300 - 47000)	--	--
	Vent	100%	1200	(350–3600)						
TDCPP	Floor	100%	2100	(580–19000)	--	--	1900	(<90 - 56000)	--	--
	Vent	100%	5600	(1200–22000)						

^1^[18]; n=20.

^2^[11]; n=20.

^3^[8]; n=50.

^4^[34]; n=13 (HBCD isomers).

^5^[16]; n=31.

Limited comparison data are available for several of the newer use flame retardants. Concentrations of BTBPE and HBCD were significantly higher in airplanes compared to U.S. homes [8,34], while concentrations of TBB and TBPH, components of the PentaBDE replacement Firemaster 550, and TDCPP, also used as PentaBDE replacement, were found at similar concentrations as recently found in homes. Additional context for the concentrations found on airplanes will be available as more research is performed on exposure to these newer use flame retardants.

### Comparison of carpet dust and vent dust

Flame retardant concentrations in dust collected from the vents were generally greater than concentrations measured in carpets. We calculated the ratio of vent dust to floor dust for each compound and for each airplane, and then calculated summary statistics. Median vent:floor dust ratios were greater than 1 for the major congeners in the PentaBDE commercial product (BDE 47 – 2.1; BDE 99 – 1.2; BDE 100 – 2.1; BDE 153 – 2.1; BDE 154 – 2.0), but unity for BDE 209 and 0.6 for BDE 183 (The inconsistent results for BDE 99 may be due to coelution issues with TBB). Median ratios for the non-PBDE flame retardants were greater than one for TDCPP (3.0), TBB (1.9), BTBPE (4.8), and TBPH (1.6), near unity for HBCD (1.0), Dechlorane Plus (1.0, 1.3), and less than unity for HBB (0.44). Dust from the vents likely represents smaller particles and might preferentially accumulate congeners that migrate to dust via volatilization, while dust from the floor likely contains larger particles that may be enriched in PBDEs that migrate to dust via abrasion. Additional research needs to be conducted to determine if this is true, and also which dust sample type is most relevant to exposure.

### Comparison by airplane manufacturer

Flame retardant concentrations differed by airplane manufacturer for some flame retardants (analyses were restricted to comparisons of the two major airplane manufacturers, Boeing and Airbus, due to limited sample size for the other three manufacturers in this study). Concentrations of the PentaBDE-associated congeners (BDEs 47, 99, 100) were significantly elevated in the Boeing airplanes compared to the Airbus airplanes, but only in the samples from the vents (p < 0.05); for BDEs 153 and 154, median concentrations in the vents were also elevated in Boeing airplanes compared to Airbus airplanes (1600 v. 630 ng/g and 1100 v. 280 ng/g, respectively) but did not reach statistical significance (p>0.05), likely due to the small sample size. Median concentrations of BDE 183 and BTBPE in the vent dust, and HBCD in floor dust, were notably higher in Airbus planes compared to Boeing planes (p > 0.05) (BDE183: 1200 v. 350 ng/g; BTBPE: 4800 v. 1300 ng/g; HBCD: 17000 v. 5100 ng/g). It is important to acknowledge that we were not able to control for age of the airplane or date of refurbishment in our analyses, which could be important confounders. In the study by Christiannsson et al., they report dust concentrations with information on the manufacturer but did not explicitly evaluate the data for differences by manufacturer [18]. We analyzed their data, presented in Table 2 of their paper, and did not find any significant differences in PBDE concentrations in dust by manufacturer.

**Table 2:** PBDE concentrations (ng/wipe) on hand-wipes from 9 flight attendants and 1 passenger compared to the general population and office workers

	**Post-flight (this study)**				**General population**^**1**^		**Office workers**^**2**^	
**Congener**	**Hand location**	**Pct detect**	**Median**	**Range**	**Median**	**Range**	**GM**	**Range**
BDE 47	Palm	100%	4.7	(4.1 - 71)	42	(<DL - 570)	33	(5.7 - 1100)
	Back	89%	3.1	(<DL- 29)				
BDE 85	Palm	89%	0.3	(<DL - 5.8)	1.3	(<DL - 37)	1	(0.2 - 59)
	Back	56%	0.3	(<DL - 2.8)				
BDE 99	Palm	100%	7.4	(3.8 - 110)	41	(0.9 - 750)	27	(4.4 - 1400)
	Back	100%	5.6	(1.6 - 54)				
BDE 100	Palm	100%	1.6	(0.9 - 22)	7.1	(0.08 - 140)	5.3	(0.9 - 260)
	Back	100%	1.1	(0.3 - 9.6)				
BDE 153	Palm	100%	0.7	(0.4 - 12)	2.6	(<DL - 290)	1.9	(0.3 - 120)
	Back	100%	0.3	(0.2 - 5.3)				
BDE 154	Palm	89%	0.5	(<DL - 7.5)	2.2	(<DL - 59)	1.7	(0.3 - 99)
	Back	67%	0.4	(<DL - 3.7)				
BDE 183	Palm	89%	0.9	(<DL - 11)	0.21	(<DL - 8.5)	0.3	(<0.1 - 8.7)
	Back	44%	0.3	(<DL - 1.7)				
BDE 209	Palm	100%	69	(25–390)	26	(<DL - 270)	12	(<1.0 - 110)
	Back	100%	43	(6.4 - 150)				

^1^ Data from [32]; n=33; samples represent total of both hands, sum of palm and back of hand.

^2^ Data from [16]; n=31; samples represent total of both hands, sum of palm and back of hand.

### Source Identification by XRF

Bromine concentrations were detected in materials of all airplane types independent of the age of the airplane (1986–2008), ranging from less than the limit of detection (approximately 10 ppm) to percent level. While it may be hypothesized that bromine concentrations on airplanes would differ by age of the airplane due to temporal changes in usage patterns of PBDEs, older planes are continually retrofitted (e.g., new carpet and seats) on a regular basis and therefore this finding was not unexpected. No significant differences were observed by airplane type or manufacturer, indicating widespread, and consistent, usage of brominated flame retardants in all airplanes.

**Figure 1:**
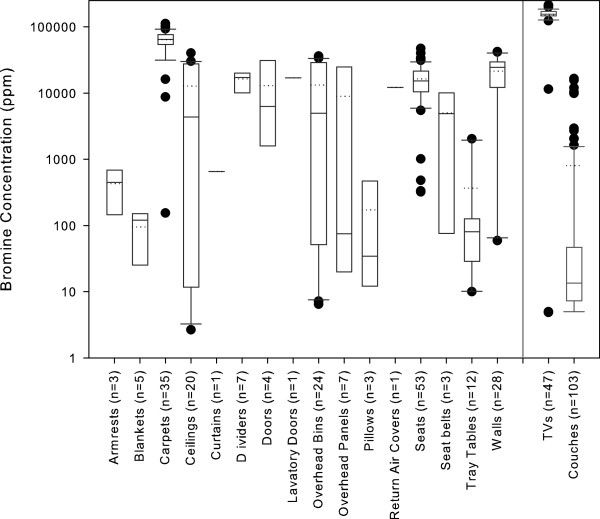
**Distribution of bromine concentrations in airplane materials in this study compared to TVs and couches[**[11]**].**

Within airplanes, bromine was identified in a majority of materials tested (Figure 1). Carpets were found to contain the highest, and most consistently elevated, concentrations of bromine. The bromine concentrations in airplane materials were similar to concentrations found in consumer products in homes, such as televisions and couches, as previously reported by Allen et al. [11] (Figure 1).

### Bromine by XRF as a predictor of PBDEs in airplane dust

**Figure 2:**
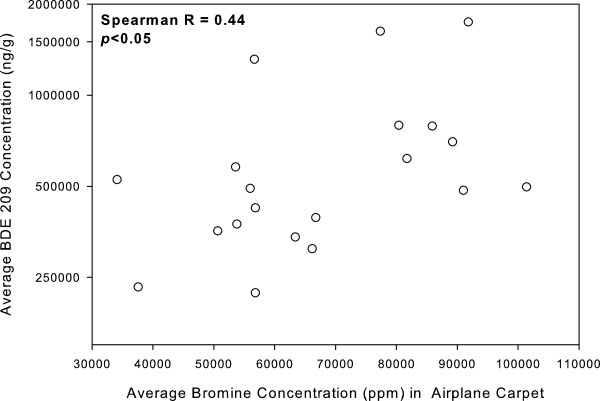
Bromine concentrations in airplane carpet (ppm) measured by XRF in comparison to average BDE 209 concentrations in dust (ng/g)].

Bromine concentrations measured in airplane carpets by XRF were significantly correlated with the BDE 209 concentration in airplane dust (Spearman R = 0.44, p<0.05) (Figure 2). This finding was specific to carpets and BDE 209; we did not observe any other significant associations between bromine content in products (e.g., seats) and PBDE concentrations in dust (e.g., BDE 47) (p>0.05). Bromine measured by XRF is a sensitive but not necessarily specific marker for brominated flame retardants, including PBDEs [11]. Therefore, the bromine measured in carpets and other materials cannot be definitively determined to be solely from PBDEs. The specificity of the XRF tool for identifying PBDE based on bromine content in products is expected to decrease over time due to PBDEs no longer being the dominant brominated flame retardant and due to the subsequent use of a wider variety of alternative brominated flame retardants. However, the results of our analysis showing an association between bromine in carpets and BDE 209 concentrations in dust suggests that BDE 209 is most likely the source of bromine in the carpets. We further explored this hypothesis by examining the relationship between bromine and antimony in carpets and other materials, knowing that antimony trioxide is often used in conjunction with DecaBDE as a synergist in a 3:1 ratio [35]; antimony trioxide is not used with PentaBDE or other brominated flame retardants, to the best of our knowledge. Bromine and antimony measured by XRF were strongly correlated in carpet samples (Spearman r = 0.78; p<0.001); a relationship that was much stronger when one influential point was removed (0.92; p<0.001). Bromine and antimony were not correlated in other materials in the airplane (e.g., Seats - Spearman r = 0.12; p=0.40).

### Hand-wipe concentrations

Post-flight PBDE concentrations from hand-wipes (ng/sample) for each congener and by sample location (palm or back of hand) are presented in Table 2, and are reported in comparison to hand-wipe concentrations from a convenience sample of 33 adults in the U.S. and 31 office workers collected using a similar methodology [2,16]. PBDE concentrations on the palm and back of hand were highly correlated within each congener (Spearman r =0.77 – 0.94, p<0.05). However, samples taken on the palm of the hand were consistently higher than the back of the hand for all congeners; the median ratio of palm:back ranged from 2.5 – 3.5, with a maximum of 8.4 (Table 2). Concentrations for congeners associated with the PentaBDE commercial product were highly correlated (palm: Spearman r = 0.81-0.99 (median 0.94), p<0.05; back of hands: Spearman r = 0.67 – 0.98 (median 0.93), p<0.05). The correlations were significant but generally weaker between the PentaBDE congeners and BDE 183 (palm: Spearman r = 0.76, p<0.05; back of hands: Spearman r = 0.69 (median), p=0.05). BDE 209 was not correlated with BDE 183 and not correlated with any of the PentaBDE congeners on the back of the hands. However, on the palm, BDE 209 was moderately correlated with all of the PentaBDE congeners except for BDE 47.

Hand-wipe concentrations for all congeners except for BDE 209 were lower in post-flight samples compared to those from the general population and office workers. For BDE 209, the concentrations were notably higher in individuals who had recently completed a cross-country (USA) flight (median: 84 v. 26 ng/sample (total of palm and back of hand)), and the maximum concentration observed was nearly two-fold higher (470 v. 270 ng/sample (total of palm and back of hand)). The consistently elevated BDE 209 concentrations in dust from all airplanes may explain the elevated hand-wipe concentrations in individuals who have recently traveled on an airplane. The data suggest that hand-wipe samples may be a useful exposure assessment tool when attempting to estimate exposure to PBDEs in locations where it is difficult to obtain dust samples (e.g., airplanes, schools).

## Conclusion

This study adds to the limited body of knowledge regarding exposure to flame retardants on commercial aircraft, an environment long hypothesized to be at risk for maximum exposures due to strict flame retardant standards for aircraft materials. Through collaboration with airline partners we were able to obtain dust measurements on active-use commercial airplanes and further characterize the dust exposure pathway for PBDEs, as well as identify novel flame retardants on airplanes. Our findings indicate that flame retardants are widely used in many airplane components and all airplane types, as expected. Most flame retardants, including TDCPP, were detected in 100% of dust samples collected from the airplanes. The concentrations of BDE 209 were elevated by orders of magnitude relative to residential and office environments. More research is needed in this area to further characterize current, and future, exposure and potential health risks related to flame retardants on airplanes.

## Abbreviations

HBCD: Hexabromocyclododecane; PBB: Polybrominated biphenyl; PCB: Polychlorinated biphenyl; PBDE: Polybrominated diphenyl ether; TDCPP: Tris(1,3-dichloro-isopropyl)phosphate; TBPH: bis-(2-ethylhexyl)-tetrabromo-phthalate (TBPH); VOC: Volatile organic compound; XRF: X-ray fluorescence.

## Competing interests

The authors declare that they have no competing interests.

## Authors’ contributions

JA designed the study, conducted the field sampling, performed the data analysis and drafted the manuscript. HS performed the laboratory analysis of the dust samples and contributed to the drafting of the manuscript. JV conducted the field sampling. EM participated in the design of the study and analysis. MM oversaw the hand-wipe sampling collection and processing. SH and CR performed the laboratory analysis of the hand-wipe samples. JS participated in the design of the study, data analysis and drafting of the manuscript. All authors contributed to, read and approved the final manuscript.

## Study design

Cross-Sectional Assessment of Flame Retardant Sources, Exposures and Pathways on Airplanes.
